# *N*,*N*′-(Ethane-1,2-diyldi-*o*-phenyl­ene)bis­(pyridine-2-carboxamide)

**DOI:** 10.1107/S1600536811042309

**Published:** 2011-10-22

**Authors:** Shuranjan Sarkar, Hong-In Lee

**Affiliations:** a Department of Chemistry, Kyungpook National University, Daegu 702-701, Republic of Korea

## Abstract

The title mol­ecule, C_26_H_22_N_4_O_2_, is centrosymmetric and adopts an *anti* conformation. Two intra­molecular hydrogen bonds, *viz.* amide–pyridine N—H⋯N and phen­yl–amide C—H⋯O, stabilize the *trans* conformation of the (pyridine-2-carboxamido)­phenyl group about the amide plane. In the crystal, the presence of weak inter­molecular C—H⋯O hydrogen bonds results in the formation of a three-dimensional network.

## Related literature

For a related structure, see: Meghdadi *et al.* (2006 ▶). For applications of the pyridine-bearing carboxamides, see: Song *et al.* (2010 ▶); Piguet *et al.* (1997 ▶); Lessmann & Horrocks (2000 ▶); Singh *et al.* (2008 ▶). For the synthesis of the ligand, see: Jain *et al.* (2004 ▶).
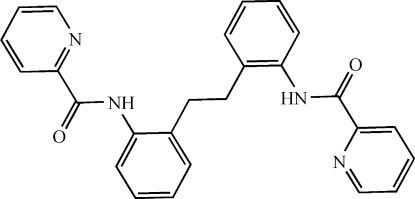


## Experimental

### 

#### Crystal data

C_26_H_22_N_4_O_2_
*M* *_r_* = 422.48
Monoclinic, 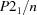

*a* = 5.9548 (5) Å
*b* = 11.9548 (10) Å
*c* = 14.8133 (12) Å
β = 91.429 (2)°
*V* = 1054.21 (15) Å^3^
*Z* = 2
Mo *K*α radiation
μ = 0.09 mm^−1^
*T* = 200 K
0.25 × 0.16 × 0.12 mm

#### Data collection

Bruker SMART CCD area-detector diffractometer
7762 measured reflections
2616 independent reflections
1118 reflections with *I* > 2σ(*I*)
*R*_int_ = 0.074

#### Refinement

*R*[*F* ^2^ > 2σ(*F* ^2^)] = 0.047
*wR*(*F* ^2^) = 0.090
*S* = 0.84
2616 reflections
149 parameters
H atoms treated by a mixture of independent and constrained refinement
Δρ_max_ = 0.19 e Å^−3^
Δρ_min_ = −0.21 e Å^−3^

### 

Data collection: *SMART* (Bruker, 2000 ▶); cell refinement: *SAINT* (Bruker, 2000 ▶); data reduction: *SAINT*; program(s) used to solve structure: *SHELXS97* (Sheldrick, 2008 ▶); program(s) used to refine structure: *SHELXL97* (Sheldrick, 2008 ▶); molecular graphics: *ORTEPIII* (Burnett & Johnson, 1996 ▶) and *CrystalMaker* (CrystalMaker, 2007 ▶); software used to prepare material for publication: *publCIF* (Westrip, 2010 ▶).

## Supplementary Material

Crystal structure: contains datablock(s) I, global. DOI: 10.1107/S1600536811042309/kp2356sup1.cif

Structure factors: contains datablock(s) I. DOI: 10.1107/S1600536811042309/kp2356Isup2.hkl

Additional supplementary materials:  crystallographic information; 3D view; checkCIF report

## Figures and Tables

**Table 1:** Hydrogen-bond geometry (Å, °)

*D*—H⋯*A*	*D*—H	H⋯*A*	*D*⋯*A*	*D*—H⋯*A*
N2—H14⋯N1	0.93 (2)	2.11 (3)	2.632 (2)	114.7 (18)
C13—H13⋯O1	0.95	2.33	2.941 (3)	122
C12—H12⋯O1^i^	0.95	2.48	3.352 (3)	152
C9—H9*B*⋯O1^ii^	0.99	2.61	3.299 (2)	127

Symmetry codes: (i) 
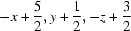
; (ii) 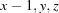.

**Fig. 1.**
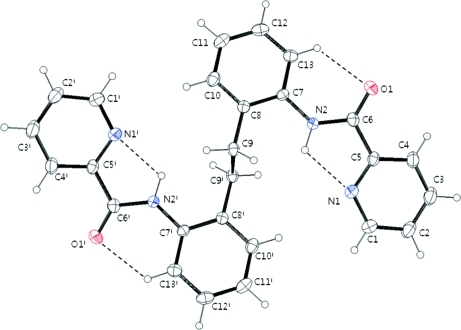
ORTEP drawing of the title compound showing 30% probability displacement ellipsoids and the atomic numbering scheme. Dotted lines represent the intramolecular hydrogen bonds. Atomic-numberings for H atoms are omitted for clarity. Symmetry code to generate the molecule: (i) -x + 1, -y + 2, -z + 1.

**Fig. 2.**
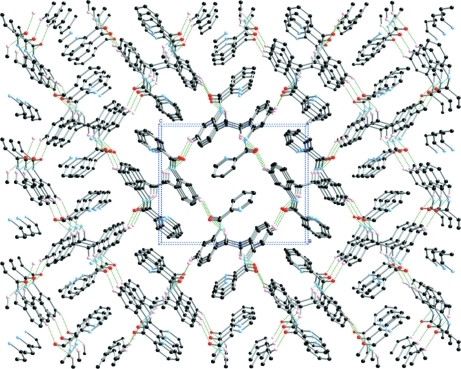
Perspective view of crystal packing along a axis. Green dotted lines represent the intermolecular hydrogen bonds. C, N, and H atoms are coloured in black, blue, and pink, respectively. All H atoms except for those related to the intermolecular hydrogen bonds are omitted for clarity.

## supplementary crystallographic information

### Comment

Pyridine-bearing carboxamide, a biologically ubiquitous functional group, is an important ligand-construction unit for coordination chemistry (Song *et al.*, 2010). This burgeoning class of ligands can be readily obtained from the condensation reaction between pyridyl carboxylic acid and amine (Jain *et al.*, 2004) and have been used to obtain a wide diversity of consequential structural motifs such as helicates (Piguet *et al.*, 1997; Lessmann & Horrocks, 2000), dinuclear and polynuclear complexes (Singh *et al.*, 2008). In order to explore coordination chemistry of bis(pyridineamide) ligands, we have synthesised the title compound (I) and report here its crystal structure.

The molecule contains two *N*-phenylene(pyridine-2-carboxiamide) moieties linked by an ethylene bridge. The crystal of (I) adopts an *anti* conformation (Fig. 1). Only a half of the molecule constitutes the asymmetric unit and the other half is centrosymmetrically related. The bond distances observed in (I) do not deviate significantly from the mean values of those in *N*,*N'*-(methylenedi-*p*-phenylene)bis(pyridine-2-carboxamide) (Meghdadi *et al.*, 2006). The angle between the phenylenyl and pyridyl rings is about 10°. Two intramolecular hydrogen bonds of N_amide_—H···N_py_ and C_ph_—*H*···O_amide_ stabilise *trans* conformation of the *N*-phenylene(pyridine-2-carboxiamide) moiety about the amide plane (Table 1 and Fig. 1). Two relatively weak intermolecular hydrogen bonds of C12—H12···O_amide_ and C9—H9b···O_amide_ present to form overall three-dimensional netowork in the crystal (Table 1 and Fig. 2).

### Experimental

A solution of 2,2'-ethylenedianiline (1.09 g, 5 mmol) in pyridine (5 mL) was added to a solution of pyridine-2-carboxylic acid (1.25 g, 10 mmol) in pyridine (10 mL). After the resulting solution was stirred at 313 K for 10 min, triphenyl phosphate (3.21 g,10 mmol) was added dropwise and the reaction mixture was stirred at 373 K for 4 h. The reaction mixture was cooled to room temperature and stirred for 10 h. The volume of the solution was reduced to 10 mL and kept over night in a hood. The remaining solution was filtered, thoroughly washed four times with 100 mL solvent of water and acetone (1:1), and dried in vacuum. X-ray quality crystals were obtained by slow diffusion of diethyl ether into a dichloromethane solution at room temperature (yield 90%; m.p. about 473 K).

### Refinement

H-atom of N—H was refined isotropically. All H-atoms at C atoms were placed in geometrically ideallized positions and constrained to ride their parent atoms with the bond lengths of 0.95 Å and 0.99 Å for aryl and methylene H atoms, repectively, and with the isotropic displacement paprameteres of *U*_iso_(H) = 1.2*U*_eq_(C).

### Crystal data

**Table:**

C_26_H_22_N_4_O_2_	*F*(000) = 444
*M**_r_* = 422.48	*D*_x_ = 1.331 Mg m^−^^3^
Monoclinic, *P*2_1_/*n*	Mo *K*α radiation, λ = 0.71073 Å
Hall symbol: -P 2yn	Cell parameters from 1330 reflections
*a* = 5.9548 (5) Å	θ = 2.8–25.8°
*b* = 11.9548 (10) Å	µ = 0.09 mm^−^^1^
*c* = 14.8133 (12) Å	*T* = 200 K
β = 91.429 (2)°	Block, colourless
*V* = 1054.21 (15) Å^3^	0.25 × 0.16 × 0.12 mm
*Z* = 2	

### Data collection

**Table:**

Bruker SMART CCD area-detector diffractometer	1118 reflections with *I* > 2σ(*I*)
Radiation source: fine-focus sealed tube	*R*_int_ = 0.074
graphite	θ_max_ = 28.3°, θ_min_ = 2.2°
phi and ω scans	*h* = −7→7
7762 measured reflections	*k* = −15→11
2616 independent reflections	*l* = −19→19

### Refinement

**Table:**

Refinement on *F*^2^	Primary atom site location: structure-invariant direct methods
Least-squares matrix: full	Secondary atom site location: difference Fourier map
*R*[*F*^2^ > 2σ(*F*^2^)] = 0.047	Hydrogen site location: inferred from neighbouring sites
*wR*(*F*^2^) = 0.090	H atoms treated by a mixture of independent and constrained refinement
*S* = 0.84	*w* = 1/[σ^2^(*F*_o_^2^) + (0.0126*P*)^2^] where *P* = (*F*_o_^2^ + 2*F*_c_^2^)/3
2616 reflections	(Δ/σ)_max_ < 0.001
149 parameters	Δρ_max_ = 0.19 e Å^−^^3^
0 restraints	Δρ_min_ = −0.21 e Å^−^^3^

### Special details

**Table:**

Geometry. All e.s.d.'s (except the e.s.d. in the dihedral angle between two l.s. planes) are estimated using the full covariance matrix. The cell e.s.d.'s are taken into account individually in the estimation of e.s.d.'s in distances, angles and torsion angles; correlations between e.s.d.'s in cell parameters are only used when they are defined by crystal symmetry. An approximate (isotropic) treatment of cell e.s.d.'s is used for estimating e.s.d.'s involving l.s. planes.
Refinement. Refinement of *F*^2^ against ALL reflections. The weighted *R*-factor *wR* and goodness of fit *S* are based on *F*^2^, conventional *R*-factors *R* are based on *F*, with *F* set to zero for negative *F*^2^. The threshold expression of *F*^2^ > σ(*F*^2^) is used only for calculating *R*-factors(gt) *etc*. and is not relevant to the choice of reflections for refinement. *R*-factors based on *F*^2^ are statistically about twice as large as those based on *F*, and *R*- factors based on ALL data will be even larger.

### Fractional atomic coordinates and isotropic or equivalent isotropic displacement parameters (Å^2^)

**Table:**

	*x*	*y*	*z*	*U*_iso_*/*U*_eq_	
N1	0.8708 (3)	0.71472 (14)	0.45440 (12)	0.0405 (5)	
C1	0.8708 (4)	0.64611 (19)	0.38332 (15)	0.0499 (7)	
H1	0.7466	0.6485	0.3418	0.060*	
C2	1.0440 (5)	0.57144 (19)	0.36728 (17)	0.0516 (8)	
H2	1.0382	0.5237	0.3160	0.062*	
C3	1.2230 (5)	0.5680 (2)	0.42681 (17)	0.0545 (8)	
H3	1.3435	0.5176	0.4174	0.065*	
C4	1.2270 (4)	0.63841 (18)	0.50077 (15)	0.0452 (7)	
H4	1.3501	0.6378	0.5428	0.054*	
C5	1.0471 (4)	0.70976 (17)	0.51194 (14)	0.0321 (6)	
C6	1.0488 (4)	0.78986 (18)	0.59027 (14)	0.0316 (6)	
O1	1.1900 (3)	0.78453 (12)	0.65163 (10)	0.0425 (4)	
N2	0.8816 (3)	0.86669 (15)	0.58299 (12)	0.0333 (5)	
H14	0.789 (4)	0.8555 (18)	0.5327 (15)	0.073 (9)*	
C7	0.8427 (4)	0.96392 (17)	0.63478 (13)	0.0282 (5)	
C8	0.6431 (4)	1.02237 (17)	0.61789 (13)	0.0282 (5)	
C9	0.4708 (4)	0.98304 (17)	0.54855 (12)	0.0308 (5)	
H9A	0.3226	1.0149	0.5632	0.037*	
H9B	0.4587	0.9006	0.5518	0.037*	
C10	0.6084 (4)	1.12198 (18)	0.66456 (14)	0.0379 (6)	
H10	0.4734	1.1627	0.6539	0.046*	
C11	0.7673 (4)	1.16257 (18)	0.72620 (15)	0.0425 (7)	
H11	0.7412	1.2308	0.7571	0.051*	
C12	0.9627 (4)	1.10390 (19)	0.74251 (14)	0.0421 (7)	
H12	1.0720	1.1318	0.7845	0.051*	
C13	1.0005 (4)	1.00386 (18)	0.69758 (13)	0.0352 (6)	
H13	1.1343	0.9627	0.7098	0.042*	

### Atomic displacement parameters (Å^2^)

**Table:**

	*U*^11^	*U*^22^	*U*^33^	*U*^12^	*U*^13^	*U*^23^
N1	0.0473 (15)	0.0353 (12)	0.0385 (12)	0.0060 (10)	−0.0035 (10)	−0.0072 (10)
C1	0.059 (2)	0.0470 (17)	0.0437 (16)	0.0059 (14)	−0.0067 (14)	−0.0121 (13)
C2	0.074 (2)	0.0395 (17)	0.0413 (16)	0.0124 (14)	0.0094 (15)	−0.0059 (13)
C3	0.068 (2)	0.0478 (18)	0.0481 (17)	0.0240 (15)	0.0113 (16)	−0.0006 (14)
C4	0.0487 (19)	0.0428 (16)	0.0442 (16)	0.0168 (13)	0.0035 (13)	0.0024 (13)
C5	0.0394 (16)	0.0260 (13)	0.0314 (13)	0.0055 (10)	0.0068 (11)	0.0065 (10)
C6	0.0353 (16)	0.0290 (14)	0.0306 (13)	0.0010 (11)	0.0038 (11)	0.0084 (11)
O1	0.0430 (12)	0.0442 (10)	0.0399 (10)	0.0075 (8)	−0.0103 (8)	0.0046 (8)
N2	0.0355 (14)	0.0330 (12)	0.0311 (12)	0.0081 (9)	−0.0053 (10)	−0.0056 (9)
C7	0.0318 (15)	0.0292 (13)	0.0239 (12)	−0.0006 (10)	0.0034 (10)	−0.0018 (10)
C8	0.0305 (15)	0.0296 (13)	0.0249 (12)	0.0001 (10)	0.0053 (10)	0.0028 (10)
C9	0.0291 (14)	0.0310 (13)	0.0324 (13)	0.0025 (10)	0.0043 (11)	0.0031 (10)
C10	0.0444 (18)	0.0344 (15)	0.0354 (14)	0.0044 (11)	0.0102 (13)	0.0012 (11)
C11	0.059 (2)	0.0331 (15)	0.0365 (14)	−0.0052 (13)	0.0141 (14)	−0.0064 (11)
C12	0.0527 (19)	0.0460 (17)	0.0278 (13)	−0.0140 (13)	0.0034 (13)	−0.0024 (12)
C13	0.0375 (16)	0.0404 (15)	0.0278 (13)	−0.0035 (11)	0.0026 (11)	0.0015 (11)

### Geometric parameters (Å, °)

**Table:**

N1—C1	1.335 (3)	C7—C13	1.390 (3)
N1—C5	1.337 (3)	C7—C8	1.396 (3)
C1—C2	1.389 (3)	C8—C10	1.395 (3)
C1—H1	0.9500	C8—C9	1.509 (3)
C2—C3	1.367 (3)	C9—C9^i^	1.543 (3)
C2—H2	0.9500	C9—H9A	0.9900
C3—C4	1.381 (3)	C9—H9B	0.9900
C3—H3	0.9500	C10—C11	1.386 (3)
C4—C5	1.382 (3)	C10—H10	0.9500
C4—H4	0.9500	C11—C12	1.375 (3)
C5—C6	1.504 (3)	C11—H11	0.9500
C6—O1	1.224 (2)	C12—C13	1.390 (3)
C6—N2	1.357 (3)	C12—H12	0.9500
N2—C7	1.415 (2)	C13—H13	0.9500
N2—H14	0.93 (2)		
			
C1—N1—C5	117.4 (2)	C13—C7—N2	121.9 (2)
N1—C1—C2	123.0 (2)	C8—C7—N2	117.7 (2)
N1—C1—H1	118.5	C10—C8—C7	118.3 (2)
C2—C1—H1	118.5	C10—C8—C9	119.8 (2)
C3—C2—C1	118.6 (2)	C7—C8—C9	121.84 (19)
C3—C2—H2	120.7	C8—C9—C9^i^	112.8 (2)
C1—C2—H2	120.7	C8—C9—H9A	109.0
C2—C3—C4	119.4 (2)	C9^i^—C9—H9A	109.0
C2—C3—H3	120.3	C8—C9—H9B	109.0
C4—C3—H3	120.3	C9^i^—C9—H9B	109.0
C3—C4—C5	118.2 (2)	H9A—C9—H9B	107.8
C3—C4—H4	120.9	C11—C10—C8	121.2 (2)
C5—C4—H4	120.9	C11—C10—H10	119.4
N1—C5—C4	123.3 (2)	C8—C10—H10	119.4
N1—C5—C6	116.96 (19)	C12—C11—C10	119.9 (2)
C4—C5—C6	119.7 (2)	C12—C11—H11	120.1
O1—C6—N2	125.7 (2)	C10—C11—H11	120.1
O1—C6—C5	122.1 (2)	C11—C12—C13	120.1 (2)
N2—C6—C5	112.3 (2)	C11—C12—H12	120.0
C6—N2—C7	130.0 (2)	C13—C12—H12	120.0
C6—N2—H14	112.8 (15)	C12—C13—C7	120.1 (2)
C7—N2—H14	116.9 (15)	C12—C13—H13	120.0
C13—C7—C8	120.4 (2)	C7—C13—H13	120.0
			
C5—N1—C1—C2	−0.1 (4)	C6—N2—C7—C8	−173.7 (2)
N1—C1—C2—C3	−0.1 (4)	C13—C7—C8—C10	0.9 (3)
C1—C2—C3—C4	−0.1 (4)	N2—C7—C8—C10	−176.24 (18)
C2—C3—C4—C5	0.4 (4)	C13—C7—C8—C9	178.45 (17)
C1—N1—C5—C4	0.5 (3)	N2—C7—C8—C9	1.3 (3)
C1—N1—C5—C6	178.20 (18)	C10—C8—C9—C9^i^	96.1 (3)
C3—C4—C5—N1	−0.6 (4)	C7—C8—C9—C9^i^	−81.4 (3)
C3—C4—C5—C6	−178.3 (2)	C7—C8—C10—C11	0.1 (3)
N1—C5—C6—O1	172.4 (2)	C9—C8—C10—C11	−177.55 (19)
C4—C5—C6—O1	−9.8 (3)	C8—C10—C11—C12	−0.4 (3)
N1—C5—C6—N2	−8.7 (3)	C10—C11—C12—C13	−0.3 (3)
C4—C5—C6—N2	169.2 (2)	C11—C12—C13—C7	1.2 (3)
O1—C6—N2—C7	8.3 (4)	C8—C7—C13—C12	−1.5 (3)
C5—C6—N2—C7	−170.7 (2)	N2—C7—C13—C12	175.5 (2)
C6—N2—C7—C13	9.2 (3)		

Symmetry codes: (i) −*x*+1, −*y*+2, −*z*+1.

### Hydrogen-bond geometry (Å, °)

**Table:**

*D*—H···*A*	*D*—H	H···*A*	*D*···*A*	*D*—H···*A*
N2—H14···N1	0.93 (2)	2.11 (3)	2.632 (2)	114.7 (18)
C13—H13···O1	0.95	2.33	2.941 (3)	122
C12—H12···O1^ii^	0.95	2.48	3.352 (3)	152
C9—H9B···O1^iii^	0.99	2.61	3.299 (2)	127

Symmetry codes: (ii) −*x*+5/2, *y*+1/2, −*z*+3/2; (iii) *x*−1, *y*, *z*.
